# Improving knowledge, attitudes, and practices on dengue and diarrhea in rural primary school students, their parents, and teachers in Colombia: A cluster-randomized controlled trial

**DOI:** 10.1371/journal.pntd.0010985

**Published:** 2022-12-27

**Authors:** Diana Sarmiento-Senior, Maria Ines Matiz, Sandra Vargas-Cruz, Juan Felipe Jaramillo, Victor Alberto Olano, Audrey Lenhart, Thor Axel Stenström, Neal Alexander, Hans J. Overgaard

**Affiliations:** 1 Instituto de Salud y Ambiente, Universidad El Bosque, Bogotá, Colombia; 2 Liverpool School of Tropical Medicine, Liverpool, United Kingdom; 3 Institute for Water and Waste Water Technology, Durban University of Technology, Durban, South Africa; 4 MRC International Statistics and Epidemiology Group, London School of Hygiene and Tropical Medicine, London, United Kingdom; 5 Faculty of Science and Technology, Norwegian University of Life Sciences, Ås, Norway; 6 Department of Microbiology & Tropical Disease Research Center, Faculty of Medicine, Khon Kaen University, Khon Kaen, Thailand; Washington University School of Medicine, UNITED STATES

## Abstract

**Background:**

Improved education on water-related diseases in schools could help to reduce disease burden. This paper presents specific results on knowledge, attitudes and practices (KAP) of a cluster-randomized controlled trial to reduce diarrheal disease and dengue entomological risk factors in rural primary schools in Colombia. The aim was to investigate whether enhanced educational interventions on dengue and diarrheal disease in schools could improve KAP scores related to these diseases in students and teachers in rural primary schools, as well as the students’ parents.

**Methodology/Principal findings:**

A factorial cluster-randomized controlled trial was carried out in 35 rural primary schools in two municipalities in Cundinamarca, central Colombia. Schools were randomized into four arms: interventions related to diarrheal disease (DIA), dengue (DEN), both (DIADEN), or no interventions (control, CON). Both educational and physical interventions to reduce risk factors of dengue and diarrhea were implemented. Comprehensive teachers’ manuals were developed and deployed to guide the learning activities. The intervention was carried out over two school years. The knowledge scores of students receiving dengue interventions (DEN, DIADEN) increased by 1.16 point score (0.75–1.56, p<0.001) and those receiving diarrhea interventions (DIA, DIADEN) increased by 1.15 point score (0.67–1.63, p<0.001). The attitude and practice scores of students receiving the diarrhea interventions increased (Attitudes: 0.41 [0.11–0.71, p = 0.01]; Practices: 0.33 [0.01–0.65, p = 0.042]), but not for those receiving the dengue interventions (p = 0.31 and p = 0.08, respectively).

**Conclusions/Significance:**

There were increases in knowledge scores among students, their teachers and their parents for both diseases. However, the attitudes and practices components were not affected to the same extent. The hypothesis that the students would disseminate knowledge acquired from the educational interventions to their parents was confirmed for dengue, but not for diarrhea.

**Trial registration:**

ISRCTN40195031 The trial is registered in the Current Controlled Trials under Infections and Infestations category.

## Author summary

The transmission of both dengue and diarrheal diseases depends on water. For dengue, the mosquitoes that transmit the infection lay their eggs in containers that hold water, and this same water may contain microorganisms that cause diarrheal diseases. Improved education on these diseases in schools could help reduce their burden. In this paper we present the results of an integrated intervention on dengue and diarrhea in terms of knowledge, attitudes and practices of students, teachers and parents in 35 rural primary schools in central Colombia. The schools were randomized into four groups: interventions related to diarrheal disease alone, dengue alone, both, or neither. For two years, both educational and physical interventions to reduce risk factors were implemented. The interventions increased knowledge scores among students, their teachers and their parents for both diseases. However, the attitudes and practices components were not affected to the same extent. There was evidence that students disseminated their new knowledge about dengue to their parents, although this was not the case for diarrhea.

## Introduction

Water-related diseases affect communities worldwide. Several of these diseases, such as *Aedes*-borne diseases (e.g., dengue) and water-borne diseases (e.g. diarrhea), may have common risk factors, such as household water storage containers. Integrated disease management targeting water, sanitation, hygiene, community waste disposal, as well as mosquito vector control, could improve community health in cost-effective ways [1].

Worldwide, about 1.9 million deaths and 123 million disability-adjusted life years (DALYs) in 2016 were associated with unsafe water supply, inadequate sanitation and hygiene [2]. In Colombia, diarrhea is a frequent cause of morbidity and mortality, with 2.8 per 100,000 inhabitants under five years of age dying from related conditions in 2017 [3]. In terms of dengue, Colombia reported the highest numbers of cases in the Andean region in 2018–2020, with an annual incidence of 174 cases per 100,000 inhabitants [4]. The main dengue vector, *Aedes aegypti* (L.), is abundant in rural as well as urban areas [5], and the next most important vector, *Aedes albopictus* (S.), is also present [6]. Other viruses transmitted by *A*. *aegypti* also circulate in the country, such as chikungunya [7] and Zika [8].

The Sustainable Development Goals (SDG) to ensure healthy lives and promote well-being (SDG 3) and to ensure inclusive and equitable quality education (SDG 4) could be simultaneously addressed by engaging schools to improve community health equity and health literacy through more engaging educational strategies. Key community problems must, therefore, be identified and actions modified through participatory social interventions applying social theories to promote healthy behaviors through individual action in social contexts [9,10]. To achieve behavioral change regarding a particular topic, and before an educational intervention is implemented, it is necessary to understand what people know about the topic, their needs and current practices. Knowledge, Attitudes and Practices (KAP) surveys are a quick way to learn about a community, their customs and health behaviors, and identify important gaps in knowledge that may influence practices [11]. On this basis, interventions have been planned and executed with varying results.

In the case of dengue, there is good evidence that community participation and mobilization can reduce infestation by the mosquito vector [12,13] and also reduce infection in some settings [14]. Although community participation has been questioned in terms of effectiveness to reduce numbers of dengue cases [15], more recent research indicates more favorable results [14,16]. Educational programs on dengue have been delivered to communities [17–19] and to primary schools [20–24] and KAP surveys have often been used as a tool to evaluate the impact of such programs. In the case of diarrheal diseases and water, KAP surveys have centered on hygiene, sanitation and water and food management [25,26] providing information about the knowledge of how communities collect and use water, their attitudes towards water storage and management, and the practices promoted by health interventions and adherence to preventive practices like boiling or handwashing.

Most of the published studies which implemented a curriculum or school program on dengue alone were carried out in urban areas [17,19,21,23,27] and a few of them evaluated the impact of a school intervention in the community [14,17,19,22,28]. There are a number of examples where integrated interventions have been implemented to make the impact stronger, for example in the case of dengue, applying participatory, chemical, physical, social and educational interventions [14,16,19,27,29]. A large study in Asia with an eco-bio-social approach found that effective control required context-specific approaches, and that schools were important breeding sites for the mosquito vectors [30]. For drinking water and sanitation improvement, some studies have evaluated combining water interventions, for example, structural changes in supply or point-of-use treatment with hygiene education interventions to reduce diarrheal disease in different settings [31,32]. Involving schools, teachers and students is particularly encouraging, for example, through school health curricula improvements, advancing general knowledge and behavioral change through student dissemination to the communities and by additional participatory initiatives [19,24,33]. The extended acknowledgement of children’s rights being more than beneficiaries of welfare services has empowered them as active individuals in society and researchers in their communities [34].

Apart from our trial, we have not found any studies on integrated interventions for dengue and diarrhea. We previously reported entomological outcomes of the trial [35], and here report KAP results. The objective of this cluster-randomized controlled trial was to establish whether enhanced educational interventions on dengue and diarrheal diseases would improve the knowledge, attitudes, and practices related to these diseases in students and teachers in rural primary schools. A secondary objective was to assess whether the knowledge, attitudes, and practices of these diseases were improved in parents of children who participated in the study arms where the educational interventions were implemented.

## Methods

### Ethics statement

The study was approved by the Comité Institucional de Ética en Investigaciones de la Universidad El Bosque, Bogotá, Colombia on 30 August 2011 (Acta No. 146) and the Ethical Review Board of London School of Hygiene and Tropical Medicine (reference number 6289). For students to participate, it was necessary for them to give written or oral assent. In addition, written or oral consent was sought from parents or guardians. In some cases, consent documentation was misplaced between the parents or guardians and the schools, but the ad-hoc ethical committee of the Universidad El Bosque (Acta No. 009 of 27/11/2014) permitted the analysis and publication of all children’s results, considering that the study was considered minimal risk under the Colombian Ministry of Health’s Resolution 8430 of 1993.

Students from grades 2 to 5 present at the school and with oral assent received and completed the KAP questionnaire. The trial is registered in the Current Controlled Trials under Infections and Infestations category (no. ISRCTN40195031).

### Setting and participants

This trial was carried out in rural primary schools in the municipalities of Anapoima and La Mesa, department of Cundinamarca, central Colombia during 2011–2014. Both municipalities are at 87 km and 74 km respectively to the west of Bogota, the capital city. Anapoima has an average altitude of 700m, a mean temperature of 26°C and 12357 inhabitants in 2014, while La Mesa’s corresponding figures are 1200m, 22°C and 27632 inhabitants. The average rainfall in both municipalities are 1300mm, with a bimodal pattern with peaks between April-May and October-November. The main economic activities are tourism, agriculture, such as raising livestock and growing sugar cane, coffee and fruit trees. A large part of the tourism consists of visitors from Bogota who stay in hotels or short term rental properties [36].

**Fig 1:**
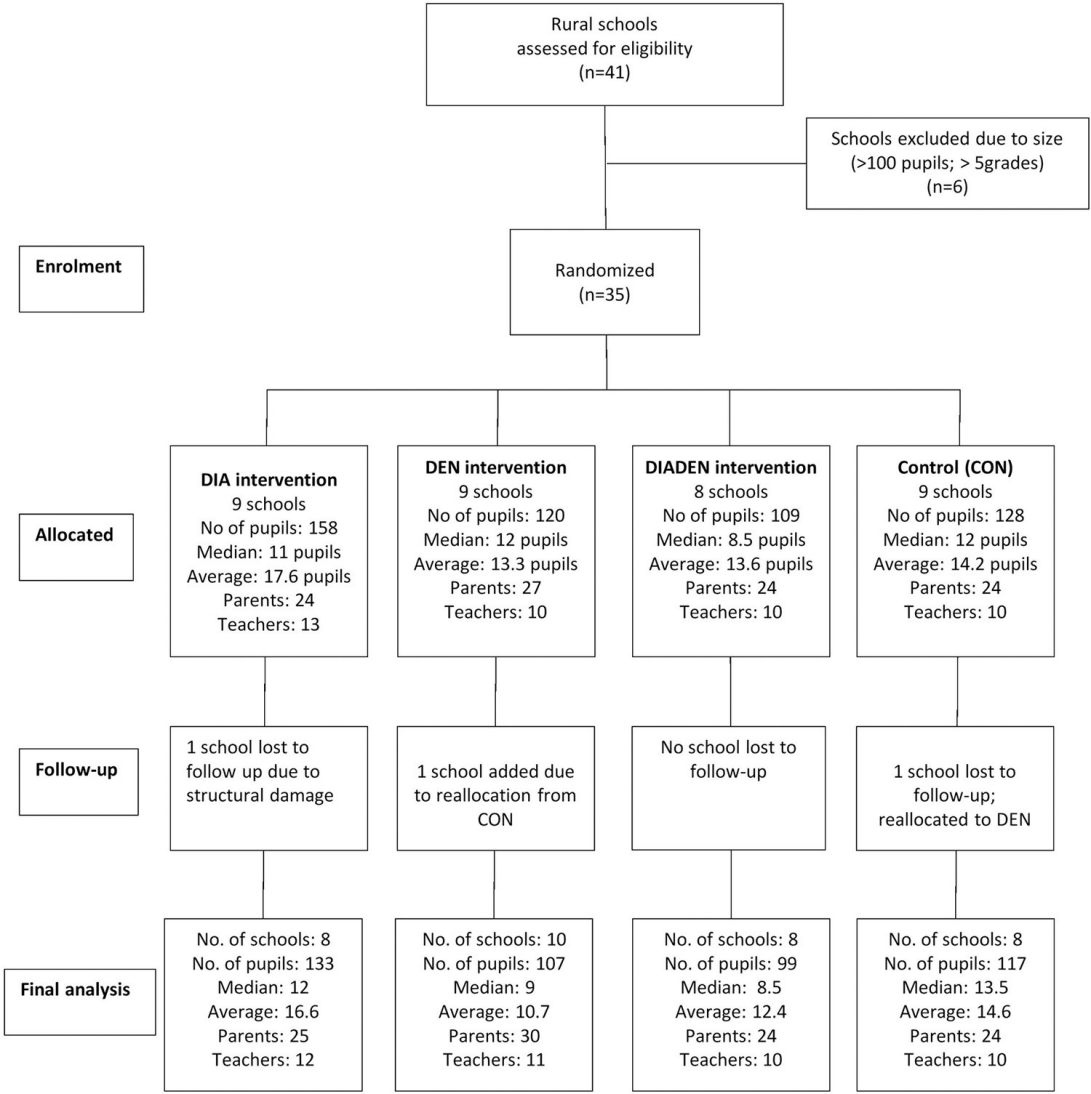
Flow diagram. Follow-up of schools and students in La Mesa and Anapoima municipalities assigned to the intervention arms. DIA = diarrhea, DEN = dengue, DIADEN = diarrhea and dengue.

All rural primary schools were assessed for eligibility to participate in the trial. Entomological and water quality outcomes of the trial have been described elsewhere [35,37]. Thirty-five rural schools were included, 17 in Anapoima and 18 in La Mesa, based on their small size (<100 students and ≤ five grades), teaching strategies and teacher-student dynamics. Within each school, all students were taught in the same classroom with differentiated spaces and tasks according to grade. Most of the schools had only one teacher, and the average number of students was around 15. These schools were not involved in any other diarrheal or dengue control program during the study. All students were eligible for inclusion. Newly enrolled children were also eligible to participate, maintaining an open cohort. Likewise, incoming teachers were eligible for inclusion. Students who moved to a school within the study area became part of the arm into which they moved. Students that moved out of the study area also left the study (Fig 1). At the beginning of the study, there were 44 teachers in the 35 schools (Fig 1). After the allocation of the interventions, one school was closed due to structural damage. Another school was reallocated to DEN from CON because it also suffered structural damage and was closed in 2012 (after the first semester of interventions the students in this school were moved to the closest available school [35]). Since the closest school was in the CON arm and the transferred students had already started receiving the DEN interventions, this school was moved to the DEN arm. After reconstruction of the first school, the students returned there, resulting in both schools remaining in the DEN arm for the duration of the study.

### Study design

This was a 2×2 factorial cluster-randomized controlled trial. The 35 rural primary schools were randomized to the four arms: three intervention arms and control (Fig 1). The allocation process was carried out as previously described (36). The trial was not blinded. A cluster design was used because the interventions are delivered at schools, with students nested within schools. A CONSORT statement is provided in the supporting information (S1 Checklist).

### Interventions

Schools were randomized to four study arms: physical and educational interventions against diarrheal diseases (DIA), dengue (DEN), both (DIADEN), or no interventions (control, CON). The interventions were implemented in February-April 2012 and continued for the scheduled duration of two school years (four school semesters) until November 2013. The two sets of interventions (DIA and DEN) consisted of physical and educational elements. Details of the physical interventions have been reported previously [35]. Briefly, the DIA physical interventions included drinking water filters, covering all drinking water storage containers, and cleaning containers once a year. The DEN physical interventions included insecticide-treated curtains, covering water storage containers (whether for drinking or not) and applying the insect growth regulator pyriproxyfen in water containers that could not be covered [38–41].

The DIA educational intervention included lessons on identification of diarrhea symptoms, transmission pathways (food, water, and hygiene), risk factors, activities for promotion of hand washing and hygiene, water and health relationships. The DEN educational intervention included lessons on identification of dengue symptoms; transmission routes; risk factors; the biology, ecology and control of vectors; and the importance of solid waste as mosquito breeding sites. These interventions were developed through the Significant Learning model [42], which emphasizes learning by doing or practicing concepts in everyday life. This method expects that practices can be modified by getting a conscious view of one’s action in the environment. Once a problem is identified through activities, there is a proper basis to introduce healthy habits and make them everyday practices.

The applied activities of the DIA intervention included a handwashing campaign with soap (before eating and after bathroom visits) and daily bathroom clean-up campaign delivered throughout the two years of interventions. The bathroom clean-up campaigns included provision of sufficient soap, a mop and cleaning liquid, verification of infrastructural conditions for hand washing. Schools without daily water supply were recommended alternative water-saving options, such as using two water bowls for washing hands, one to wet hands before applying soap and another to rinse without immersing hands, then changing the water when it looked grey or muddy and using it for flushing toilets; or asking students to bring their own boiled water to school in a thermos. The applied activities of the DEN intervention included a weekly school clean-up campaign consisting of identification and elimination of potential vector breeding sites and solid waste collection around the school.

### Educational material

Eight teachers’ manuals were developed to guide learning activities for the students. There were two sets of educational material, one for dengue and one for diarrhea, each set consisted of four manuals. The teachers’ manuals had information on relevant items of each disease and instructions to lead the activities adjusted to the school children’s age (5 to 15 years old). The educational interventions, for both DIA and DEN, followed three processes: design, application, follow-up and evaluation with constant feedback from teachers. During the two-year program, all eight educational manuals were designed and produced for each intervened school (i.e., all other than those in the control arm). Educational guides, flyers about hand hygiene and other parts of the intervention were provided to control schools after the trial. In the first year, the manuals contained information and activities and in the second year included mainly playful activities for the students related to each theme.

### Outcomes

The primary outcomes were students’ scores on knowledge, attitudes and practices about diarrhea and dengue assessed as averages on a scale from 0 to 10. The secondary outcomes were scores for teachers and students’ parents on knowledge, attitudes and practices about diarrhea and dengue, again assessed as averages on a scale from 0 to 10. These were secondary outcomes because they measure effects of the educational intervention in populations which were only indirectly exposed to the intervention.

### Sample size

The sample size was calculated—in terms of numbers of schools and numbers of students per school—based on disease outcomes, because these, rather than the KAP outcomes reported here, were primary for the project as a whole [35].

### Data collection

KAP questionnaires were administered to students, teachers and parents (Table 1).

**Table 1:** Data collection times, number of questions and number of individuals surveyed for students, teachers, and parents. Teacher and parent questionnaires also included questions about number of rooms in the school or the house, hygiene and sociodemographic characteristics, etc.

Group	Data collection	Time	Total no. of questions	Knowledge questions		Attitudes questions		Practices questions		Individuals surveyed
				Dengue	Diarrhea	Dengue	Diarrhea	Dengue	Diarrhea	
Students	Baseline	2011	14	3	3	1	1	2	4	515
	Final	2013	76	18	21	7	10	8	12	456
Teachers	Baseline	2012	75	10	5	10	8	4	7	43
	Final	2013								43
Parents	Baseline	2012	122	9	6	7	6	6	11	99
	Final	2013								103

### Students

Baseline data collections were done in all schools in 2011 using a KAP questionnaire adapted from our previous study [43]. Separate questionnaires for each of the KAP components were developed for evaluating the impact of the educational interventions on students, teachers and students’ parents. A new student questionnaire was developed and validated during October 2012 and May 2013 with the support of experts in pedagogy and graphic design. Multiple choice questions in this questionnaire were presented as problem situations with three illustrated choices for answer, most of them in the knowledge component. Attitudes and practices questions used three point Likert scales with options never, sometimes and always. This questionnaire was applied twice, in May 2013 and in October 2013, about 1–1.5 years after the interventions began. Bearing in mind the literacy level of these students, the assessments were done in four grades, from second to fifth grades. The assessments were done during school time following an established protocol. For students in the second and third grades, the survey was done in groups of up to five students. One questionnaire was delivered to each student, then a member of the research team read the questions and each possible answer, avoiding prompting for the correct ones. After the reading of each question and its options, each student had a couple of minutes to select their answer in the questionnaire. For students in the fourth and fifth grades, each recorded their own answer in the presence of a member of the research team.

### Teachers

For parents and teachers, the questions were adapted from DANE (National Administrative Department of Statistics) national census [44] and international WASH surveys [45]. The same questionnaire was used at baseline in 2012 and post-intervention in 2013. A total of 43 teachers took part in both data collections. Of the original 43, nine had been replaced by the second data collection (21% turnover). Multiple-choice questions were used for knowledge and practices, and Likert scale questions for attitudes and practices. In addition to the 44 KAP questions about the diseases, the questionnaire also included 31 questions about socioeconomic characteristics of the teachers and dissemination of information about the diseases.

### Parents

A total of 99 and 103 students’ households were surveyed in 2012 and 2013, respectively. Three households for each school were randomly selected and one parent per household was administered the parent KAP questionnaire, which had 122 questions in total. In addition to the 45 KAP questions about dengue and diarrhea, the questionnaire included 77 items in an observational checklist about the household’s sanitary and hygiene conditions, water supply, socioeconomic conditions of the household and water management practices.

### Statistical methods

To calculate the total score for each of the three components (KAP) for each disease and for each of the three groups (students, teachers and parents), the points per question were added. Each question was classified according to the dimension it belonged (knowledge, attitudes, practices), and then grouped by themes. For example: sanitation; acknowledging water and food contamination, acknowledging preventive practices to avoid contamination of water and food, acknowledging the diarrheal disease, vector identification and habits, identification of breeding sites, acknowledging dengue symptoms and management of the disease, identification of transmission routes, prevention of the disease.

A point was assigned to each theme to have the same weight in the scale, whether composed of one or more questions. Questions of attitudes and practices dimensions used a three-point Likert scale with the options: always, sometimes and never for each situation enunciated. The score reflected the desirability of the situation. For example, to assess the attitude towards self-medication, a question was: can we take pills without the doctor’s prescription? The scoring of the answer was as follows: never 1 point, sometimes 0.5 points and always 0 points. The maximum points available varied between questions, so they were expressed on a scale from 0 to 10.

The average KAP scores for each school were calculated over all surveys done in the intervention period (including the first and second rounds). These school-level averages were the response variable for a factorial analysis of covariance similar to the one described previously [35], but using linear regression rather than negative binomial regression. The explanatory variables were i) whether or not the school received the dengue interventions, ii) whether or not it received the diarrhea interventions, and iii) municipality, by which the randomization was stratified. This means that, for example, the effect of the dengue interventions is estimated simultaneously by comparing both the DEN arm versus the control (CON) arm, and the combined (DIADEN) arm versus the DIA arm [46]. Aggregating the data to the school level accounts for the clustering which results from randomizing by school.

## Results

### Baseline characteristics

Data on the altitudinal range, entomological indices, water sources and water quality of the schools have been reported before [37]. There were only minor baseline differences between schools, although those in the DEN and CON arms were located at a higher average altitude than schools in the other arms. A higher proportion of schools in the DIADEN arm used rainwater as the main water source, implying the lack of daily water supply for hygiene tasks such as washing hands and bathrooms.

### Students

In 2011, 515 rural school students in the 35 schools were assessed for their baseline KAP score for each disease [43], with little variation between the arms (Table 2).

**Table 2:** General baseline school characteristics and baseline knowledge, attitudes and practices (KAP) scores of students in 35 rural primary schools distributed across four arms before the interventions in Anapoima and La Mesa municipalities, Colombia in 2011. KAP scores were assessed on a scale from 0 to 10.

Variable	DIA	DEN	DIADEN	CON
Number of schools	9	9	8	9
**General**				
Students per arm of intervention	158	131	109	117
Average of students per school	17.6	13.3	13.6	14.2
Median of students per school	11	12	8.5	12
Male/female ratio	1.1	1.1	1.2	2.0
Mean student age (in years) (SD)^a^	9.4 (0.5)	9.4 (0.6)	9.2 (0.6)	9.4 (0.4)
Age range	6–14	7–16	7–14	6–14
Total no. of teachers	13	10	10	10
**Baseline values of primary outcome variables (SD)** ^a^				
**Dengue**				
Mean Knowledge score in students per school	6.3 (1.5)	6.0 (1.2)	6.8 (1.5)	6.1 (1.4)
Mean Attitudes score in students per school	9.5 (1.2)	9.0 (0.8)	8.3 (1.8)	9.6 (0.5)
Mean Practices score in students per school	4.7 (1.2)	4.7 (1.8)	5.9 (2.1)	5.0 (1.3)
**Diarrhea**				
Mean Knowledge score in students per school	6.7 (1.0)	7.1 (1.2)	7.8 (1.3)	6.7 (0.9)
Mean Attitudes score in students per school	8.8 (0.8)	8.4 (1.4)	9.0 (1.2)	9.1 (1.0)
Mean Practices score in students per school	6.8 (0.6)	7.0 (1.3)	6.8 (1.1)	6.1 (1.4)

^a^ Standard deviation (SD) calculated across schools.

### Teachers

There were 34 women and 9 men aged between 25 and 61 years old (mean = 39.8, SD = 8.9). Only 7 teachers (15%) lived outside the study area in neighboring municipalities and 13 (30%) had been living less than five years in Anapoima or La Mesa. Most (86%) had higher education degrees (18 bachelor, 19 postgraduate).

### Parents

Of the 103 heads of households interviewed, 76 (74%) were males, 26 (25%) were females and one (1%) did not provide any information. Their age range was 20–83 years old (mean = 41.9, SD = 12.5). The main levels of education were 31% with incomplete primary school, 36% completed primary school, and 15% incomplete secondary school. The family size ranged between 2 and 11 members (mean = 4.4) and 59 families (57%) had four or fewer members. Generally, one household consisted of one family, but in four households (4%) there were three or more families.

### Outcomes

A total of 457 students (261, 57.1% males) in second to fifth grade were surveyed. Their ages were between 6 and 15 years (median = 9.4; SD = 1.74). There were 260 (57%) students in schools in La Mesa municipality and 197 (43%) in Anapoima. The first round of the survey included 457 students who answered the dengue questionnaire and 456 who answered the diarrhea questionnaire. In the second round, the corresponding numbers were 457 and 458.

### Primary outcomes

**Fig 2:**
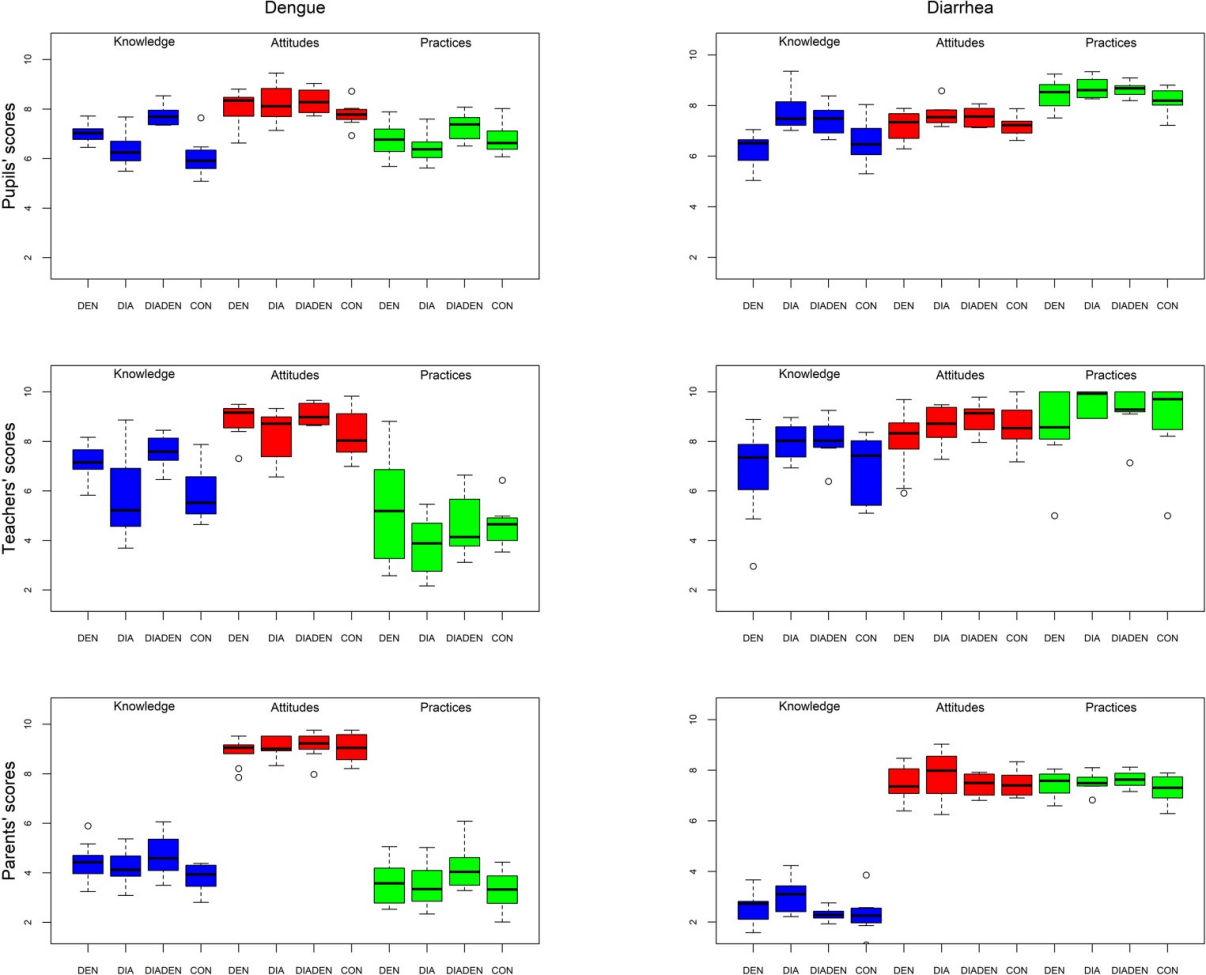
Mean scores of knowledge, attitudes and practices (KAP) of students (students), teachers and parents in the intervention period. KAPs related to diarrhea (left panel) and dengue (right panel) are shown for the four intervention arms (DEN, DIA, DIADEN, CON) in rural primary schools in Colombia, 2012–2013. KAP scores were assessed on a scale from 0 to 10.

The overall KAP scores in the intervention period for students for each disease are shown in Fig 2. The educational interventions on dengue increased students’ knowledge of the disease in those arms that received them (DEN, DIADEN) relative to those that did not (Table 3). There were no significant differences between arms in the attitudes and practices components. The educational interventions on diarrhea increased students’ knowledge, as well as attitudes and practices in intervention arms (DIA, DIADEN) compared with the control.

**Table 3:** Students. Effect of interventions on dengue (DEN and DIADEN arms) and diarrhea (DIA and DIADEN arms) on students’ knowledge, attitudes and practices related to dengue and diarrhea. KAP scores were assessed on a scale from 0 to 10.

Disease	Component	Intracluster correlation coefficient	Effect of intervention (difference in point score)	95% confidence interval	*p*
Dengue	**Knowledge**	**0.174**	**1.16**	**0.75–1.56**	**<0.001**
	Attitudes	0.080	0.18	-0.17–0.53	0.306
	Practices	0.057	0.4	-0.05–0.84	0.079
Diarrhea	**Knowledge**	**0.153**	**1.15**	**0.67–1.63**	**<0.001**
	**Attitudes**	**0.028**	**0.41**	**0.11–0.71**	**0.01**
	**Practices**	**0.061**	**0.33**	**0.01–0.65**	**0.042**

### Secondary outcomes

#### Teachers

The overall KAP scores in the intervention period for teachers for each disease are shown in Fig 2. For dengue, there were statistically significant effects only in the knowledge and attitudes outcomes (Table 4). For diarrhea, knowledge was the only one of the three outcomes that showed a statistically significant effect of the intervention.

**Table 4:** Teachers. Effect of interventions on dengue (DEN and DIADEN arms) and diarrhea (DIA and DIADEN arms) on teachers’ knowledge, attitudes and practices related to dengue and diarrhea. KAP scores were assessed on a scale from 0 to 10.

**Disease**	**Component**	**Effect of intervention (on the scale of 0 to 10)**	**95% confidence interval**	***p***
Dengue	**Knowledge**	**1.55**	**0.81–2.29**	**<0.001**
	**Attitudes**	**0.72**	**0.15–1.29**	**0.015**
	Practices	0.7	-0.34–1.74	0.179
Diarrhea	**Knowledge**	**1.19**	**0.28–2.09**	**0.012**
	Attitudes	0.49	-0.18–1.16	0.148
	Practices	0.68	-0.17–1.54	0.112

### Parents

The overall KAP scores in the intervention period for parents for each disease are shown in Fig 2. Parents of the students in the dengue intervention arms (DEN, DIADEN) showed higher dengue knowledge relative to the control arm (Table 5). There was a possible positive effect on dengue practices in parents whose children belonged to the dengue intervention arm, although this effect was not statistically different from parents whose children did not receive the intervention (p = 0.06). There were no significant differences in knowledge, attitudes or practices in parents whose children experienced the diarrhea educational interventions (DIA, DIADEN) compared to the control.

## Discussion

The objective of this cluster-randomized controlled trial was to establish whether enhanced educational interventions on dengue and diarrheal diseases would improve the knowledge, attitudes, and practices related to these diseases in students and teachers in rural primary schools. A secondary objective was to assess whether the knowledge, attitudes, and practices of these diseases improved in parents of children who participated in the study arms where the respective educational interventions were implemented. Teachers’ manuals specifically developed for the project were additional outputs which could be used or adapted to other Spanish speaking countries.

**Table 5:** Parents. Effect of interventions on dengue (DEN and DIADEN arms) and diarrhea (DIA and DIADEN arms) on parents’ knowledge, attitudes and practices related to dengue and diarrhea.

Disease	Component	Effect of intervention (on the scale of 0 to 10)	95% confidence interval	*p*
Dengue	**Knowledge**	**0.56**	**0.09–1.03**	**0.021**
	Attitudes	-0.03	-0.4–0.34	0.865
	Practices	0.52	-0.02–1.06	0.06
Diarrhea	Knowledge	0.17	-0.23–0.58	0.388
	Attitudes	0.13	-0.34–0.6	0.578
	Practices	0.19	-0.11–0.49	0.212

## Students

The dengue intervention improved the students’ knowledge about dengue. The rural schools included in this study already had low to medium levels of dengue knowledge prior to the intervention [43]. Students who received the dengue intervention knew that *Aedes aegypti* was the principal vector and were able to recognize it among pictures of other mosquito species, and to identify the main breeding sites in their area (laundry sinks, uncovered water tanks). They also had a slightly higher ability to recognize disease alarm signs, compared to those who did not receive the dengue intervention: their average score was 1.16 points higher, which is about 10% of the range of the ten-point scale. The educational intervention lasted for two years and there were no other educational campaigns or local programs to control dengue during this period, indicating that the intervention described here had a significant impact on dengue knowledge. A playful intervention designed for dissemination of information from teachers to children in the 6–14-year age group in a rural context was a starting point for building interest and acceptance in students. Although the dengue intervention improved students’ knowledge, there was no evidence of an improvement in attitudes and practices. This may be because some of the dengue practices promoted in the intervention were in principle feasible for the children to do, e.g., washing water tanks or treating tanks with pyriproxyfen, but were in practice not possible for them to carry out because these practices were the responsibility of others. For example, there was a recommendation to go to the medical center when febrile, but decisions to do this are not made only by the students. Most research on the effect of education on dengue knowledge and control has been done as part of community-based approaches, including mobilization of schools as one of several community groups [12]. Several cluster randomized studies showed that such school programs reduce various dengue larval indices [19,47,48] and even dengue infection rates in children [14]. A reduction of the Breteau Index in the intervention schools in our study could have been a combined result of the physical interventions [35] as well as the educational intervention presented here. However, it is difficult to attribute how much schools contributed to the observed effects, since the interventions were community-based and not specifically school-based only. A systematic review of the effect of dengue educational interventions in schools on health and risk behaviors showed a lack of randomized controlled trials [24]. Out of 21 studies reviewed, 16 were quasi-experimental before-after designs, but only one used systematic sampling and four used random sampling of schools and children [24]. The conclusion was that using educational games, cartoons, observations, lectures and engaging students in information campaigns were effective in improving students’ knowledge, attitudes, and self-efficacy for dengue prevention and control [20,49–51]. The review also found that few studies evaluated the sustainability of protective behaviors and recommended that innovative health behavior research is needed. The sustainability and institutional adoption of the current trial was evaluated two years after the trial ended [52]. The physical, as well as the educational, interventions were assessed as moderately sustainable, whereas institutional empowerment, adaptive flexibility, financial support, etc. were considered unsustainable. The failure of institutional adoption was due to a lack of integration of the interventions into the school activities and support from the municipalities.

Results from KAP questionnaires are commonly used to evaluate dengue interventions, although results may be difficult to analyze and interpret especially in relation to how sociodemographic factors affect results. Applying multiple correspondence analysis, Higuera-Mendieta and co-authors found that interventions to improve knowledge about dengue were most effective in groups with low and middle initial dengue knowledge and that these associations were weaker in groups with higher knowledge [53]. They also found that preventive practices were not correlated with sociodemographic variables.

Although the baseline diarrhea KAP scores in students in our study were already at a medium to high level, the diarrhea intervention improved their knowledge, attitudes and practices scores regarding diarrhea. Students learned and understood the fecal-oral transmission route, how water can become contaminated, signs of alarm of dehydration and ways to prevent diarrhea from contaminated water and food. Most water, sanitation and hygiene (WASH) interventions in schools focus on the impact of school infrastructural improvements on health outcomes, school absence and behavioral change, but there are relatively few randomized studies assessing how targeted educational interventions impact health-related KAP outcomes. A recent review of the literature on WASH interventions in schools in low-income countries included 13 studies (32% of all reviewed) that assessed changes in WASH knowledge, attitudes and hygiene behaviors among students [54]. It seems most educational interventions also included infrastructural components (provision of improved handwashing facilities, soap, toilet cleaning material, etc.), and training (e.g., in handwashing). All 13 studies showed improved knowledge in WASH-related diseases and improved hygiene habits. However, there was little evidence that the school-based WASH interventions improved knowledge in family members and the larger community. Similarly, we did not detect improved KAP scores in parents following the educational intervention, unlike what was detected following the dengue intervention. It has also been argued that knowledge assessments and self-reporting of handwashing practices are not valid measures, because respondents tend to say what they think is a desirable answer, rather than to declare their practices [55]. There is a need to develop standardized methodologies to assess the results of interventions on personal hygiene education.

The intervention increased the student scores of all three KAP components for diarrhea, whereas for dengue only the scores of the knowledge component increased. This could be explained by diarrhea being easier to identify and more common than dengue. Even though the study was done in a dengue endemic area, inhabitants do not generally acknowledge the disease and sometimes interpret symptoms as a cold [43,56]. Moreover, practices to prevent diarrhea such as washing hands, drinking clean water and daily cleaning of toilets, had fewer barriers for students to execute them. Diarrhea interventions are more individual based, e.g., “I have to wash my hands before eating and after going to the toilet”. Therefore, the more frequent experiences and exposure to diarrheal diseases and its causes than with dengue and its causes might make it easier for students to internalize knowledge and transform it into habits. However, integrated interventions where both diseases can be targeted should be the focus of future arboviral and waterborne disease control and research [1].

### Teachers

Some of the teachers in the study were from other parts of the country and were initially unaware of topics such as identification of potential breeding places of the dengue vectors, mosquito appearance, and the principal disease symptoms and preventive measures. The teachers, therefore, seemed to benefit from the intervention increasing their general knowledge and attitudes about dengue. The application of the strategy throughout two years increased teachers’ knowledge of dengue and resulted in a change in their attitudes towards prevention and control activities.

Teachers also increased their knowledge related to diarrheal disease. They acknowledged dehydration warning signs, preventive practices such as covering and washing water tanks, good hygiene practices, and water treatment. Diarrhea is a common disease and is frequently included in discussions with parents in schools and at medical care centers, so the teachers’ improved knowledge could have benefited the community, although this does not seem to have been commonly observed in other studies [54]. The intervention did not affect teachers’ practices scores for either disease. Practices related to diarrheal disease were already high, indicating that teachers were aware of this common disease. Furthermore, when scores are already high it is difficult to increase scores even further. As some teachers were not from the study area and being unfamiliar with dengue, they might not have reflected that some practices designed for the school would also be applicable in their home.

### Parents

Dengue knowledge in parents whose children received the dengue intervention was higher than in parents whose children did not receive the intervention. Our program targeted students, but results showed that knowledge also disseminated to their parents. The knowledge transfer could happen by parents learning from and helping their children with the practical exercises included in their disease education to identify potential risks in and around their homes. According to personal communication with teachers, parental engagement in schools is not common and usually restricted to their children’s educational progress; therefore, it is more likely that parents learned directly from their children. Although it might not be meaningful to compare KAP scores between students, teachers and parents due to differences in questionnaires, it is noteworthy that the K and P scores for parents, especially for dengue, were conspicuously low compared to those of students and teachers (Fig 2). The educational levels of parents in our study were low, with 67% having primary education (31% incomplete, 36% complete), which could have influenced the results. Previous studies have found associations between level of education and dengue knowledge and preventive practices [53,57]. Our study found some popular misconceptions, such as dengue mosquitoes preferring to bite at night, or that both female and male mosquitoes bite humans, which might reflect that dengue prevention and information campaigns are less commonly implemented in rural areas [27].

There was a clear distinction between diarrhea practices and dengue practices in parents, and partly in teachers as well (Fig 2). The higher diarrhea scores compared with dengue could be explained by hygiene and cleaning practices that people acknowledge as important, without necessarily linking them to the disease. It has been suggested that the use of chemical interventions for dengue vector control may give a false sense of security [13,58], but more comprehensive community-based interventions have proved to be effective [12,14]. Also, good knowledge of dengue does not necessarily translate to good practices, and to raise awareness of the risks in a population, routine information, education and communication are key factors [10].

Even when people have learned how to boil water and wash hands in a proper way, if they do not have a permanent supply of water then they need to store it, potentially creating risks of contamination and vector breeding sites [59]. For both diseases, education remains a fundamental intervention that can mobilize communities around a public problem and create low cost and sustainable solutions to protect themselves against risk factors. However, governmental support is key to cover basic needs of rural areas.

### Limitations

Although the utility of the KAP approach has been questioned [60], it can yield internally consistent and reliable results if developed rigorously [61]. Also it is valuable that complementary studies with qualitative methodologies are included to have the perspectives of the participant communities since the design of the interventions and understand their inner dynamics. Including communities around the schools in the interventions could have had a larger impact. Even though this would not strictly be a school intervention, the school could still play a role in leveraging the interventions into the communities, for example through supporting stronger parent-teacher associations and parent school involvement. Including schools in community-based interventions seems to be widely implemented in both dengue and WASH research [24,54]. We do not think there was much contamination between study arms because of the relatively large distance between schools, so that it is not likely that people in the control arm would get information from people in the other arms. Some interventions on dengue in schools, such as covering of tanks or administration of pyriproxyfen, were not the responsibility of students but required the presence of an adult. It is possible that students did not take into account such activities when asked about their attitudes and practices which, in turn, could have diluted the apparent effect of the intervention.

Another possible limitation is that structural problems with the premises of two schools due to landslides led to them being re-allocated between the arms [35]. Hence the analysis is not completely intention to treat. However, the causes for re-allocation are unlikely to be associated with the original allocation. The sample size of teachers was not very large, but it proved sufficient to show differences in some endpoints, and with fairly narrow confidence intervals for these effects and the others for which no difference was established. The students’ questionnaires were not the same for the baseline as they were for the follow ups, but this should affect only statistical efficiency, not validity.

Questionnaires to assess knowledge and behavioral changes should be well adapted for the setting and the data to be collected. In our study, each questionnaire was assessed for validity and piloted to evaluate comprehension and clarity. Attitudes and practices should preferably be evaluated by observation, or with other tools, but this makes it harder to measure and also introduces biases, e.g., dependence on the particular observer.

## Conclusions

This is the first trial to combine educational, physical, and applied activities to improve knowledge, attitudes and practices to reduce dengue risk factors and diarrheal disease in rural areas. The findings are likely to be generalizable to rural schools in other countries in northern South America, and possibly beyond. Despite an observed effect on the knowledge of students, their teachers and parents, the attitudes and practices components were not affected to the same extent. The teachers supported the implementation of the program because it could easily link prevention themes with existing curricula. Furthermore, we hypothesized that the students would be able to disseminate the knowledge they acquired from the educational interventions to their parents, and this was detected for dengue, but not for diarrhea.

## Supporting information

S1 Checklist CONSORT Checklist. (PDF)
